# The relationship between social support and dimensions of elder maltreatment: a systematic review and Meta-analysis

**DOI:** 10.1186/s12877-023-04541-6

**Published:** 2023-12-18

**Authors:** Behnaz Marzbani, Erfan Ayubi, Majid Barati, Parvaneh Sahrai

**Affiliations:** 1 Department of Public Health, School of health, Hamadan University of Medical Sciences, Hamadan, Iran; 2 Cancer Research Center, Hamadan University of Medical Sciences, Hamadan, Iran; 3 Department of Public Health, School of Health, Autism Spectrum Disorders Research Center, Hamadan University of Medical Sciences, Hamadan, 6517838695 Iran

**Keywords:** Social support, Elder maltreatment, Elderly, Neglect, Meta-analysis, Systematic

## Abstract

**Introduction and aims:**

Many studies have investigated the relationship between social support and the prevention of elder abuse; however, their results are somehow inconsistent in terms of the association. This systematic review and meta-analysis aimed to investigate the published studies on the relationship between social support and the prevention of elder maltreatment.

**Materials and methods:**

An electronic search was conducted until January 2023, using such databases as PubMed, Scopus, and Web of Science. The present research included cross-sectional, longitudinal, and case-control studies. Study selection, data extraction, and risk of bias assessment were conducted by two researchers independently. The Newcastle-Ottawa checklist was utilized to evaluate the quality of studies. The random effects model was employed to perform a meta-analysis.

**Results:**

In total, 32 studies were included in this systematic review, out of which 26 articles were eligible for meta-analysis. The results showed that 68.75% of the studies were of high quality, and there is a significant relationship between social support and elder maltreatment. Accordingly, the lack of social support increased overall maltreatment (odds ratio: 1.24, 95% confidence interval: 1.16–1.33; I^2^ = 92.3%, *p* = 0.000)). Moreover, lack of social support had an increasing effect on the level of psychological abuse (1.55, 1.18–2.04; 88.7%, *p* = 0.000), physical abuse (1.31, 0.42–4.11; 76.3%, *p* = 0.005), and neglect (2.02, 0.86–4.72; 87.9%, *p* = 0.000), which shows heterogeneities among the results of the included studies. On the contrary, the lack of social support showed a decreasing effect on financial abuse (0.92, 0.70–1.21; 62.1%, *p* = 0.022).

**Conclusion:**

This systematic review provides evidence that social support in the form of structural or functional support may plays an important role in improving the quality of life of the elderly.

**Supplementary Information:**

The online version contains supplementary material available at 10.1186/s12877-023-04541-6.

## Introduction

The phenomenon of aging is one of the most sensitive periods of human life across the world, and the elderly population is growing remarkably [1, 2]. It is predicted that by 2050, the global population aged 60 and over will double and reach about 2.1 billion people [3]. With the rapid growth of the world’s elderly population, mistreatment of the elderly is becoming a growing social problem [4].

Elder maltreatment is an important public health issue with serious social, economic, and health consequences [5]. According to the definition by the World Health Organization (WHO), elder abuse is “a single or repeated act, or lack of appropriate action, which causes harm or distress to an older person” [6–8]. Despite the high rate of elder abuse, its actual rate is not known and is less reported [9, 10]. Based on the results of a systematic review of the five continents, the prevalence of elder abuse has been estimated from 2.2 to 79.75% [11]. According to the WHO definition, health refers to “complete physical, mental, and social well-being and not merely the absence of disease or infirmity” [12]. According to this definition, social health as one of the four dimensions of health plays a significant role in the quality of life of elderly people and indicates the importance of the social dimension of human beings [13].

Social support refers to the help or support provided to an individual by the members of social networks [14], and it has been defined differently: “the number of people in the participating network”, “an indicator of overall satisfaction with social support”, “the availability of multiple forms or types of support (e.g., informational/emotional, instrumental/tangible and affectionate)”, and “positive social interaction” [15, 16].

Studies have shown that higher levels of social support help to improve the quality of life related to the physical and mental health of the elderly [17] and life satisfaction [18]. The literature review indicates the high prevalence and increasing trend of misbehavior with the elderly. Although the studies conducted in this regard show the protective role of social support in reducing maltreatment, there is no consensus on the strength of the relationship between social support and maltreatment. There are even studies demonstrating that there is no connection between these two factors. Therefore, it seems necessary to summarize studies in this regard seems necessary to be able to make accurate and valid judgments about the role of social support. Therefore, the present study aimed to examine the relationship between the dimensions of misbehavior and social support through a systematic review and meta-analysis.

## Materials and methods

This systematic review and meta-analysis were conducted based on the PRISMA checklist. The research population includes all scientific articles on the relationship between social support and the dimensions of elder maltreatment, which were indexed in such databases as Web of Science, Scopus, and PubMed. In order to preserve all the valuable data, all articles published in English from the beginning to January 2023 were extracted by two independent researchers. The search strategy was developed using Medical Subject Headings (MeSH) and related keywords. The potential articles were identified by combining “social support”, “dimensions of maltreatment”, and “the elderly”. The search strategy was limited to the documents in English.

### Study selection

**Fig. 1:**
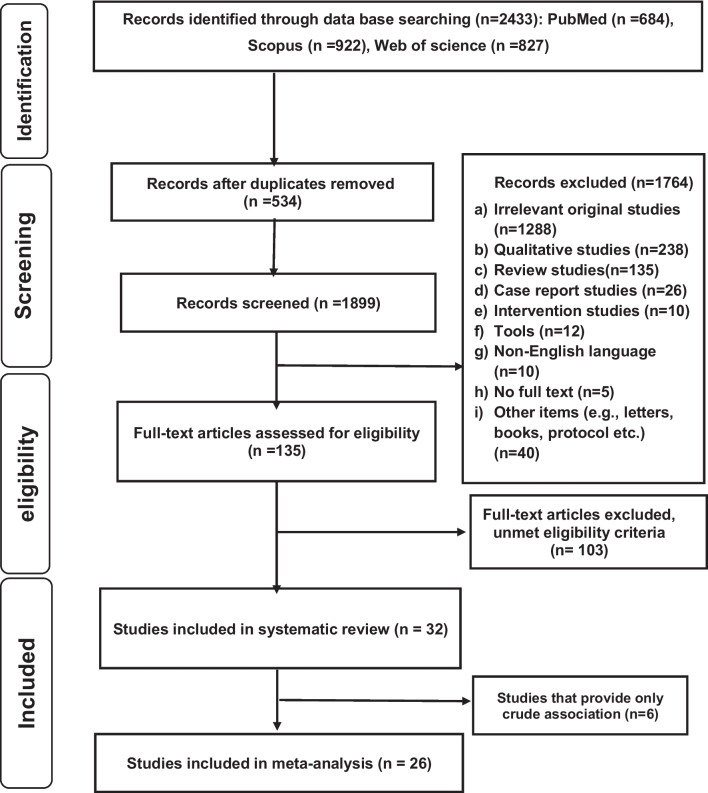
Study selection and exclusion according to the PRISMA flow diagram

The results of initial searches were independently screened by two authors (BM and PS) according to titles, abstracts, and full texts. In all stages, any disagreement among the researchers regarding the exclusion or inclusion of articles in the study was resolved through discussion and finally with the opinion of the third researcher (MB). All searched articles in the initial search were entered into EndNote X7.5 software (Fig. 1).

### Eligibility criteria

The inclusion criteria were 1) articles published in English from the beginning to January 2023, 2) types of observational studies (cross-sectional, case-control, and cohort), 3) the study population (male and female elderly people), and 4) studies with association measures, such as odds ratio (OR) with confidence interval (CI) and type of determinants (social support and types of maltreatment).

On the other hand, interventional and qualitative studies, articles with no reviews, and letters to the editor were excluded from the study owing to the lack of use of primary data. Moreover, studies the abstracts of which had no information were also removed from the research procedure.

### Data extraction and quality assessment

The information extracted from the studies in the analysis and recorded in Excel software included the type of study, year of publication, first author, study population, gender, country, number of participants, the questionnaire used, results related to social support, the outcome of the study: dimensions of maltreatment (e.g., physical, financial, psychological, sexual, and neglect), and the results of the studies. After examining the aims of the studies and the inclusion criteria, the eligible studies were evaluated in terms of methodological quality using the Newcastle-Ottawa (for observational studies). This tool examined the quality of studies considering selection, comparability, and results. It should be mentioned that two independent researchers evaluated the quality of the articles at all stages. According to this scale, the articles were scored from zero (the weakest study) to nine (the strongest). Studies with a score higher than six were considered high quality**.**

### Statistical analysis

The information of individual studies and corresponding pooled measure are summarized in forest plots. Between studies heterogeneity was evaluated using the I^2^ value and substantial heterogeneity was set as I^2^ >  50%. Random-effects meta-analysis was used to estimate pooled associations. Publication bias was checked using funnel plot and Begg’s test and Egger’s test. Meta-analysis was performed using and Stata 11 (StataCorp, College Station, TX, USA).

## Results

### Results of the searched studies

Figure 1 illustrates the process of selecting the presented articles. A total of 2433 articles were retrieved by searching three international databases. After removing 534 duplicate articles, 1899 studies remained. Of these, 1764 articles were removed after reviewing the titles and abstracts. Afterward, the full texts of 135 articles were evaluated, and after a careful review of the literature, 103 articles were excluded due to not meeting the inclusion criteria. The relationship between social support and the dimensions of maltreatment was investigated in 32 articles as the main or secondary aim, and six articles were not included in the meta-analysis due to the lack of reporting the OR index [19–24].

It should be mentioned that only 26 studies reported OR with a confidence interval that entered the meta-analysis. In total, 10 articles were removed due to repetitive content and re-reporting of information in the form of a new article [25–34]. Furthermore, six articles were excluded due to the difference in the type of index extracted despite the use of similar data, among which two studies [35, 36] had different target groups (urban and rural elderly) and four studies [37–40] reported different aspects of maltreatment.

### Study characteristics

Table 1 tabulates the characteristics of the articles. The sample size varied from 128 [20] to 26,229 [47] Participants. These studies were geographically diverse and included 22 countries (three joint studies in several countries) [37, 38, 53] in five WHO regions, including Africa [42], America [21, 22, 24, 39, 40, 43, 48, 50, 52, 53, 55, 56, 60], Europe [37, 38, 44, 45, 54, 57, 58], Southeast Asia [35, 36, 51], and Western Pacific Ocean [19, 20, 23, 41, 47, 49, 59].

**Table 1:** Main Characteristics of Studies Included in the Systematic Review and Meta Analyses

First author Year country	Sample size	Female %	Elder Abuse		Mean ± SD or prevalence (%)	Social support		Measure of association
			Type of Abuse	Tool and its description		Tool and its description	Social support scale	
Zhao et al., 2022 Chinese [19]	511	53.62	Self-neglect	ESNA-24 item (ranged from 0 to 48)	Mean (SD) 13.65 ± 10.65	Social Support Revalued Scale	Per a unit increase	β = − 0.15 (− 0.27, − 0.09)
Xu et al., 2022 China [41]	452	35.40	Self-neglect	AGSS (ranged from 0 to 24 and > =4 is defined as having self-neglect abuse)	30.30	SSRS	Per a unit increase	OR = 0.96 (0.91, 1.01)
Okojie et al., 2022 Nigeria [42]	913	–	Elder abuse Rural	Researcher-made questionnaire	14.70	–	Poor vs. adequate	OR = 3.55 (1.64, 7.69)
			Elder abuse urban		13.30		Poor vs. adequate	OR = 2.25 (1.00, 5.03)
			Psychological abuse (rural)		86.20		–	
			Psychological abuse (urban)		84.20		–	
			Sexual abuse (rural)		3.40		–	
			Sexual abuse (urban)		9.20		–	
Santos et al., 2022 Brazil [43]	159	76.73	Violence	H-S/EAST 15 items (A score of three or more points indicates increased risk for violence)	72.10	MOS-SSS Emotional/informational support	Per a unit increase	OR = 0.95 (0.91, 0.98)
Dias et al., 2022 Portugal [44]	677	63.22	Elder abuse	CTS and UK survey of elder abuse/ neglect 52 items First: frequency) never, once, twice, 3–5, 6–10, 11–20 or more than 20 times during the past year) Second: reported any abuse = Abuse	23.90	MSPSS	Low vs. high	OR = 3.96 (1.22, 12.83)
			Psychological abuse	11 items	19.90		–	
			Physical abuse	17 items	2.50		–	
			Financial abuse	9 items	5.80		–	
			Sexual abuse	8 items	1.90		–	
Simmons et al., 2021 Sweden [45]	607	44.48	One form of victimization	NorAQ (Yes or no)	7.74	One item from the annual Swedish national public health survey	No vs. yes	OR = 2.15 (0.94, 4.93)
			Any violence		26.35		–	
			Emotional violence		12.52		–	
			Physical violence		19.60		–	
			Sexual violence		7.74		–	
Oetzel et al., 2021 New Zealand [20]	128	75.78	Elder Abuse	Validated scale from previous empirical research 4 items (Ranged from 0 to 16)	Mean (SD) 0.15 ***±*** 0.26	perceived and desired social support	Per a unit increase	Correlation = − 0.18
Sooryanarayana et al., 2020 Malaysia [46]	3466	42.46	Elder abuse	questions adapted from the National Irish Prevalence Survey on Elder Abuse 30 items (Yes or no)	9.00	11-item Duke’s Social Support Index	Poor vs. good	OR = 5.03 (2.25, 11.21)
			Neglect abuse	4 items ≥10 occurrences < 10 (if it was perceived as being severe by the older person).	7.50		–	
			Financial abuse	8 items	0.80		–	
			Psychological abuse	7 items	0.70			
			Physical abuse	8 items	0.20		–	
			Sexual abuse	3 items	0.10		–	
Koga et al., 2020 Japan [47]	26,229	53.93	Multiple abuse	Researcher-made questionnaire 3 items	12.30	Researcher-made questionnaire (Emotional support)	Yes vs. no	OR = 0.89 (0.75, 1.06)
			Psychological abuse	1 item: ranging from 1 (never), 2 (once or twice), 3 (occasionally), or 4 (frequently)	11.11		Yes vs. no	OR = 0.86 (0.72, 1.03)
			Financial abuse	1 item: (Yes or no)	1.45		Yes vs. no	OR = 0.92 (0.61, 1.39)
			Physical abuse	1 item: ranging from 1 (never), 2 (once or twice), 3 (occasionally), or 4 (frequently)	1.26		Yes vs. no	OR = 1.14 (0.69, 1.89)
Zheng et al., 2019 USA [48]	3157	–	Elder abuse	H-S/EAST 10-item (Yes or no)	15.01	scale, adapted from the Health and Retirement Study	Negative vs. positive	OR = 1.51 (1.41, 1.61)
Park et al., 2018 South Korea [49]	10,674	56.80	Emotional abuse	1-item (Yes or no)	9.40	1-item (none, 1–2, 3–5, or more than 6 persons)	No vs. > 6 people	OR = 1.71 (1.37, 2.12)
Williams et al., 2017 USA [39]	5776	60.20	Multiple abuse	A series of questions elder abuse in survey form validated 25-item (yes or no)	1.70	modified five-item version of the MOS-SSS	Low vs. high	OR = 1.64(1.00, 2.67)
			Emotional abuse	4-item	27.10		–	
			Physical abuse	3-item	6.90		–	
			Sexual abuse	3-item	3.40		–	
			Neglect	6-item	33.50		–	
			Financial abuse	9-item	34.50		–	
Vilar-Compte et al., 2017 USA [50]	526	100.00	Elder abuse	GMS (22-item) (yes or no)	33.40	OSS-3 intermediate and strong social support, a dichotomous variable	Strong vs. intermediate	OR = 0.57 (0.34, 0.94)
			Physical abuse	5-item	3.80		–	
			Psychological abuse	6-item	30.60		–	
			Neglect	4-item	5.10		–	
			Financial abuse	5-item	8.40		–	
			Sexual abuse	2-item	1.10		–	
Liu et al., 2017 USA [21]	386	70.90	Financial abuse	OAFEM 79 FE statements (ranged from 0 to 158) Not event happened = score 0 Suspected of occurrence = score 1 Event happened = score 2	200	ISEL (12-item short version)	Per a unit increase	β = −.022
Nisha et al., 2016 India [51]	200	55.50	Elder abuse	EAST and two items on neglect and two additional items on financial abuse **(Present or absent)**	16.00	–	Absent vs. present	OR = 6.1 (1.8, 20.2)
			Verbal abuse		12.50		–	
			Neglect		11.00		–	
			Financial abuse		8.50		–	
			Physical abuse		1.50		–	
Melchiorre et al., 2016 Europe [37]	4467	57.30	Multiple abuse	Violence based on the UK study on elder abuse and CTS ^(^52 items^)^ (Yes or no)	22.10	MSPSS	Per a unit increase	OR = 0.98 (0.97, 0.99)
			Physical abuse	17 items	2.70		–	
			Sexual abuse	8 items	0.70		–	
			Financial abuse	9 items	3.80		–	
			Psychological abuse	11 items	19.40		–	
			Injury	7 items	0.70		–	
Beach et al., 2016 USA [52]	903	73.30	Financial abuse	adapted from work by Quinn and Tomita 4 items (Yes or no)	3.50	ISEL	Per a unit increase	OR = 0.94 (0.88, 0.99)
Chokkanathan et al., 2015 India [35]	897	–	Elder abuse	CTS (15 items) (Yes or no)	21.00	modified version of the Duke Social Support and Stress Scale MOS-SSS	Per a unit increase	OR = 0.84 (0.75, 0.95)
			Psychological abuse	3 items	19.20			
			Financial abuse	3 items	12.70			
			Neglect	2 items	12.40			
			Physical abuse	6 items	12.30			
			other	1 item	–			
Guedes et al., 2015 Canada, Albania, Colombia and Brazil [53]	1995	52.13	Psychological abuse by their family	HITS (4 items) (Ranged from 4 to 20)	9.67	measured social support using six questions	Low vs. high	OR = 1.29 (0.77, 2.17)
James et al., 2013 USA [22]	639	76.80	Scams	Susceptibility to Scams Scale (5 items) (ranged from 5 to 35)	Mean (SD) 2.88 ***±*** 0.70	MSPSS Social support	Per a unit increase	β = −0.07 (0.67, −0.81)
LEE et al., 2014 South Korea [23]	1023	82.80	Self-neglect	Subscale (SSEA) (5 items) (ranged from 5 to 35)	22.80	LSNS-6	Per a unit increase	β = −.155
Adamczyk et al., 2013 Poland [54]	518	61.50	Any type of Violence (Women)	Researcher-made questionnaire 4 items (Yes or no)	63.40	Social Support List 12 – Interactions Scale: (Higher social support >scores median scale low social support <= scores median scale)	Low vs. higher	OR = 1.93 (0.98, 3.82)
			Any type of Violence (Men)	4 items (Yes or no)	36.60		Low vs. higher	OR = 1.35 (0.46, 3.93)
			Psychological Violence (Men)	1 item (Yes or no)	30.20		Low vs. higher	OR = 0.71 (0.15, 3.62)
			Psychological Violence (Women)	1 item (Yes or no)	69.80		Low vs. higher	OR = 2.64 (0.99, 7.09)
			neglect (Men)	1 item (Yes or no)	38.10		Low vs. higher	OR = 0.51 (0.13, 1.96)
			Neglect (Women)	1 item (Yes or no)	61.90		Low vs. higher	OR = 2.51 (1.02, 6.15)
			Financial violence (Men)	1 item (Yes or no)	32.00		High social limitations	OR = 0.20 (0.03, 1.40)
			Financial violence (Women)	1 item (Yes or no)	68.00		High social limitations	OR = 0.38 (0.12, 1.17)
			Physical violence (Women)	1 item (Yes or no)	69.20		–	
			Physical violence (MEN)	1 item (Yes or no)	30.80		–	
Macassa et al., 2013 Europe [38]	4467	57.29	Overall psychological abuse (Female)	UK study of abuse/neglect of older people and CTS (11 item) (Minor or severe)	19.40	MSPSS	per a unit increase	OR = 0.98 (0.98, 0.99)
			psychological abuse (Male)		20.30		–	
Dong et al., 2013 USA [55]	404	34.65	Elder abuse (Urban)	VASS Hwalek & Sengstock (Yes or no)	31.22	SSI	Per a unit increase Low Social support	OR = 1.11 (1.04, 1.19)
			Elder abuse (Rural)		44.44		Low Social support	OR = 1.19 (1.08, 1.31)
Yan et al., 2012 USA [56]	937	42.37	Physical abuse	CTS (violence) (Total or Severe)	2.50	social support scale from the FNS	Low Social support	OR = 0.14 (0.02, 0.76)
			Sexual abuse		1.20		Low Social support	OR = 0.29 (0.03, 2.45)
			Psychological abuse		36.10		Low Social support	OR = 1.16 (0.77, 1.76)
Cevirme et al., 2012 Turkey [57]	452	46.00	Elder abuse	Researcher-made questionnaire 5 items (Yes or no)	28.50	MSPSS	Per a unit increase	OR = 0.76 (0.70, 0.82)
			Emotional abuse		57.40			
			Physical abuse		14.70			
			Economic abuse		27.90			
Naughton et al., 2011 Ireland [58]	2021	55.00	Multiple abuse	financial abuse and neglect (UK and NY prevalence studies) and CTS 29 items	2.20	Oslo-3	Poor vs. strong	OR = 3.11 (1.29, 7.46)
			Financial abuse	8 items (Yes or no)	1.30			
			Psychological abuse	7 items (ranged from 10 > = and /or if < 10)	1.20			
			Physical abuse	9 items (Yes or no)	0.50			
			Sexual abuse	2 items (Yes or no)	0.05			
			Neglect	3 items (Yes or no)	0.30			
Wu et al., 2011 China [59]	2000	–	Multiple abuse	VASS	36.20	Researcher-made questionnaire of social support	practical support from family	OR = 1.28 (1.01, 1.63)
			Physical abuse		4.90			
			Emotional abuse		27.30			
			Neglect		15.80			
			Financial Exploitation		2.00			
Amstadter et al., 2011 USA [60]	902	59.90	Emotional abuse	Researcher-made questionnaire on elder abuse 4 items (Yes or no)	5.10	MOS-SSS	low vs. high	OR = 3.51 (1.63, 7.53)
			Physical abuse	3 items	1.80		low vs. high	OR = 8.14 (0.8, 83.26)
			Neglect	6 items	5.40		low vs. high	OR = 6.74 (1.54, 29.62)
			Financial abuse	6 items	6.60		low vs. high	OR = 1.77(0.71, 4.42)
			Sexual abuse	2 items	0.30		–	
Acierno et al., 2010 USA [40]	5777	60.20	Emotional abuse	Researcher-made questionnaire on elder abuse 4 items (Yes or no)	4.60	MOS-SSS	Low vs. High	OR = 3.17 (2.14, 4.69)
			Physical abuse	3 items	1.60		low vs. high	OR = 2.95 (1.19, 7.30)
			Sexual abuse	3 items	0.60		low vs. high	OR = 5.68 (1.30, 2.44)
			Neglect	6items	5.10		low vs. high	OR = 4.14 (2.34, 7.35)
			Financial abuse by family (Use of social services)	10 items	5.20		low vs. high	OR = 0.75 (0.57, 0.98)
Choi et al., 2008 USA [24]	370	–	Self-neglect	PSA (reports of alleged abuse, neglect, and self-neglect a year.) 7 items	22.20	(number of family members and friends who may be able to help)	No. of social support	β = 0.35 (0.70, 0.002)
			Financial abuse	5 items	38.40		No. of social support	β = 0.72 (1.29, 0.15)
			Multiple abuse	30 items	51.90		–	
			Physical abuse	1 item	10.50		–	
			psychological abuse	1 item	10.50		–	
			Sexual abuse	1 item	1.00		–	
			Self-endangering behaviors	4 items	22.70		–	
			Environmental hazards	5 items	27.30		–	
			Inability to manage finances	6 items	62.20		–	
Chokkanathan et al., 2005 India [36]	400	49.50	Elder abuse	Two subscales on verbal and physical abuse taken from (CTS) and based on extant literature (two items on neglect and two items on financial abuse) (total: 18 items)	14.00	MOS-SSS	Per a unit increase	OR = 1.07 (1.04, 1.09)
			verbal abuse	8 items: frequency (at least 10 times)	10.80			
			financial abuse	2 items: (0 times, 1–2 times, 3–5 times, > 5 times)	5.00			
			physical abuse	6 items: (0 times, 1–2 times, 3–5 times, > 5 times)	4.30			
			neglect	2 items: frequency (at least 10 times)	4.30			

Elder Self-Neglect Assessment (ESNA)

Abrams Geriatric Self-Neglect Scale (AGSS)

Social Support Rate Scale (SSRS)

Hwalek-Sengstock Elder Abuse Screening Test (H-S/EAST)

Medical Outcome Study: Social Support Scale (MOS-SSS)

Multidimensional Scale of Perceived Social Support (MS-SSS)

MDS-HC: Minimum Data Set –Home Care

Conflict Tactic Scales (CTS)

NorVold Abuse Questionnaire (violence) NorAQ

Geriatric Mistreatment Scale

three-item Oslo scale (social support) (OSS-3)

Older Adult Financial Exploitation Measure (OAFEM)

Interpersonal Support Evaluation List (ISEL)

Elder Abuse Screening Test (EAST)

UK Study of Abuse and Neglect of Older People: Qualitative Findings

short instrument for Domestic violence (DV) screening (HITS scale)

Screening Scale for Elder Abuse (SSEA)

Lubben Social Network Scale (family network and friend network) (LSNS-6)

Vulnerability to Abuse Screening Scale (VASS)

Social Support Instrument (SSI)

Family Needs Screener (Risk factors for IPV) (FNS)

short version of the Personal and Relationships Profile (PRP)

Social Support Scale (Oslo-3)

After reviewing, 20 articles [20, 24, 35–37, 39, 42–48, 50, 51, 54, 55, 57–59] reported maltreatment in general. Other studies investigated some dimensions of maltreatment, such as neglect [19, 23, 24, 35, 36, 39, 41, 46, 50, 51, 54, 57–60], sexual [24, 37, 39, 40, 42, 44–46, 50, 56, 58, 60], verbal [36, 51], physical [24, 35–37, 39, 40, 44–47, 50, 51, 54, 56–60], and financial [21, 24, 35–37, 39, 40, 44, 46, 47, 50–52, 54, 57–60], as well as psychological abuse dimensions [22, 24, 35, 37–40, 42, 44–47, 49, 50, 53, 54, 56–60].

Social support was also evaluated using such different indicators as the size of the social network [21–23, 45, 48, 49, 52, 53], emotional support [43, 47], informational support [19, 43], instrumental support [19, 40, 47, 59], and social support using special scales [20, 24, 35–39, 41, 42, 44–46, 50–52, 54–58, 60].

The results also showed that in addition to the relationship between social support and the dimensions of maltreatment, some articles investigated other dimensions, including the mediating role of social capital [19, 45, 47], domestic violence [53, 54, 56], the ecological framework of the elder abuse image [37], as well as direct and indirect effects of social support on maltreatment [35]. The majority of the studies used standard tools. The most widely used tools for evaluating maltreatment and social support were the tactical scale of conflict, the multidimensional scale of perceived social support, and the medical consequences: the social support scale, in descending order.

### Quality of studies

All 32 included studies were quantitative (cross-sectional = 31 and case-control = 1) and in English. Furthermore, 22 studies were considered high quality [19, 22, 24, 35–41, 44–50, 53, 54, 56, 58, 60]. Due to the lack of access to the full text of the article, the quality assessment of the two studies was not completed [42, 59]. Table 2 tabulates the evaluation of the quality of articles.

**Table 2:** Results of the quality assessment

Author, year (Study)	Design	Items			Total NOS stars
		Selection	Comparability	Outcome/exposure	
Choi, 2008 [24]	**Case controls**	***	**	**	*******
Zhao, 2022 [19]	**Cross sectional**	****	**	*	*******
Xu, 2022 [41]	**Cross sectional**	***	**	**	*******
Santos, 2022 [43]	**Cross sectional**	***	**	*	******
Dias, 2022 [44]	**Cross sectional**	****	**	**	********
Simmons, 2021 [45]	**Cross sectional**	***	**	**	*******
Oetzel, 2021 [20]	**Cross sectional**	**	**	**	******
Sooryanarayana, 2020 [46]	**Cross sectional**	****	**	**	********
Koga, 2020 [47]	**Cross sectional**	****	**	**	********
Zheng, 2019 [48]	**Cross sectional**	****	**	**	********
Park, 2018 [49]	**Cross sectional**	****	**	*	*******
Williams, 2017 [39]	**Cross sectional**	****	**	**	********
Vilar-Compte, 2017 [50]	**Cross sectional**	****	**	**	********
Liu, 2017 [21]	**Cross sectional**	**	**	**	******
Nisha, 2016 [51]	**Cross sectional**	*	**	**	*****
Melchiorre, 2016 [37]	**Cross sectional**	****	**	**	********
Beach, 2016 [52]	**Cross sectional**	****	**	*	*******
Chokkanathan, 2015 [35]	**Cross sectional**	****	**	**	********
Guedes, 2015 [53]	**Cross sectional**	****	**	**	********
James, 2014 [22]	**Cross sectional**	****	**	**	******
Lee, 2014 [23]	**Cross sectional**	***	**	**	*******
Adamczyk, 2013 [54]	**Cross sectional**	****	**	**	********
Macassa, 2013 [38]	**Cross sectional**	****	**	**	********
Dong, 2013 [55]	**Cross sectional**	**	**	**	******
Yan, 2012 [56]	**Cross sectional**	***	**	**	*******
Cevirme, 2012 [57]	**Cross sectional**	**	**	**	******
Naughton, 2011 [58]	**Cross sectional**	****	**	**	********
Amstadter, 2011 [60]	**Cross sectional**	****	**	**	********
Acierno, 2010 [40]	**Cross sectional**	***	**	**	*******
Chokkanathan, 2005 [36]	**Cross sectional**	***	**	**	*******

### Systematic review results

Table 1 showed a total of 32 studies were included in this systematic review, among which 81% of the studies had a sample size of 400 or more and 28.12% of the studies were published from 2020 onwards. The results of 64.81% of studies have indicated that there is a significant relationship between social support and maltreatment, and 91.42% of these studies have reported the protective role of social support in reducing maltreatment with the elderly. Overall, the protective role of social support on maltreatment was estimated at 53.12%, and the highest and lowest (15.62 and 3.12%) protective roles were related to the neglect and sexual dimensions of maltreatment, respectively. In addition, among the studies that did not report a significant relationship between social support and maltreatment, 31.57% were associated with the emotional dimension of maltreatment.

six studies have examined social support as a quantitative outcome, and the rest of the studies have reported it as categories. Among the quantitative studies, four studies have demonstrated the existence of a significant negative relationship between social support and maltreatment [19–21, 23], and one study reported a positive relationship between social support and financial maltreatment and neglect [24]. In addition, no significant association was found in the study [22].

Two cross-sectional studies [40, 56] have investigated the effect of social support on sexual misconduct with random sampling; however, they were not included in the meta-analysis and were therefore included in the systematic analysis. The results of a study [40] revealed that the elderly who suffered from low social support were more likely to be subjected to sexual abuse odds ratio OR = 5.68(1.30, 2.44). However, another study [56] did not report any significant relationship between social support and sexual abuse OR = 0.29 (0.03, 2.45).

### Meta-analysis results

**Fig. 2:**
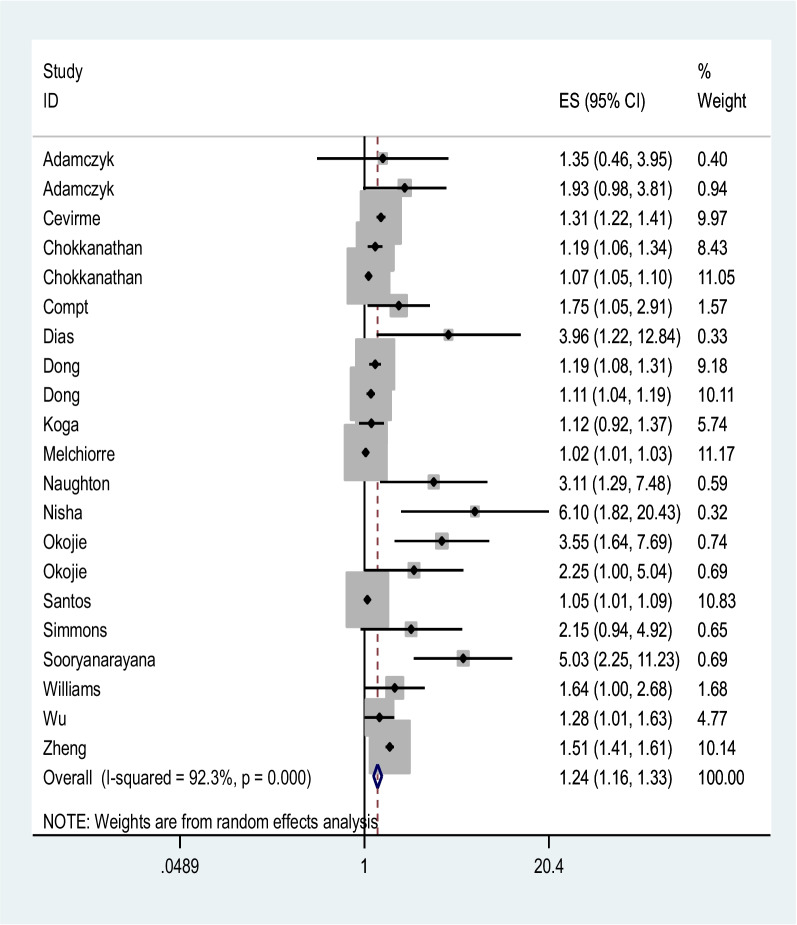
Meta-analysis of low social support and risk of multiple abuses in elderly

From some studies, two OR values have been included in the meta-analysis (Table 1). The results of the present study show that the lack of social support has increased the chance of all kinds of elder maltreatment by 24%. The meta-analysis estimation was obtained with a confidence interval of 1.16 and 1.33, which was statistically significant. The value of the I^2^ statistic in this analysis was obtained at 92.3%, which indicates the high heterogeneity of the results of the included studies (Fig. 2). Begg’s test, *p* = 0.573 and Egger’s test, *p* = 0.255, which shows that there is no publication bias for financial abuse.

**Fig. 3:**
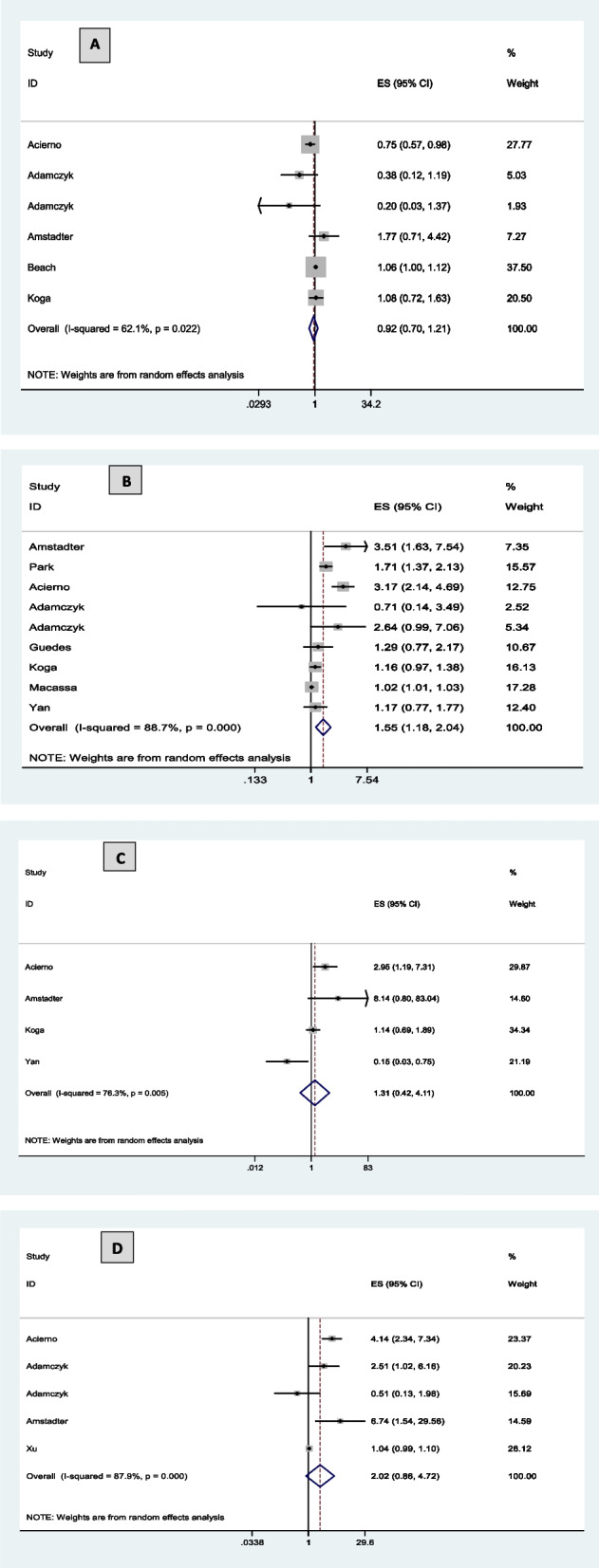
Meta-analysis of low social support and dimensions of abuse in the elderly: **A** financial abuse. **B** psychological abuse. **C** physical. **D** neglect

The relationship between social support and dimensions of maltreatment is presented in Fig. 3. The lack of social support had an increasing effect on the chances of psychological (OR = 1.55 [1.18 and 2.04]) and physical (OR = 1.31 [0.42 and 4.11]) maltreatment, as well as neglect (OR = 2.02 [0.86 and 4.72]) (Fig. 3B, C, D, respectively). On the contrary, lack of social support had a decreasing effect on financial maltreatment (OR = 0.92 [0.70 and 1.21]) in the elderly, and it was not statistically significant (Fig. 3A). Figure 3A, B, C, D illustrate the values of I^2^ statistic that are 62.1, 88.7, 76.3, and 87.9%, respectively, which indicates the heterogeneity of all the results of the included studies. The meta-analysis estimate for sexual maltreatment in the elderly was not investigated due to the limited number of data [40, 56]. Because the number of included studies on different dimensions of abuse was less than 10, the publication trend was not checked.

## Discussion

This systematic review was conducted based on a comprehensive search strategy to better recognize the relationship between social support and dimensions of elder abuse. In total, 32 studies were included in this review, of which 26 articles were eligible for meta-analysis. The results of the study show that the lack of social support had increasing effects on maltreatment in general and all dimensions. Moreover, it had the greatest impact on the dimension of neglect (neglect by others and self-neglect).

Lack of social support increases the chance of elder abuse by 24%, which was similar to the results of some studies [36, 37, 43]; however, it was not consistent with the findings of other studies [35, 44, 51]. One of the reasons for the similarity of the relationship in the study [37] is that it indicated the relationship using an ecological framework. This may reveal the low level of social support and social isolation as important risk factors for male elder abuse. In addition, some cultural social norms, as well as mass media encourage tolerance and a kind of “normalization” towards violence. Furthermore, the lack of social support from friends weakens the self-efficacy or inner strength of the elderly and makes them more vulnerable to the experience of Elder Maltreatment [48]. This shows that the differences are not only cultural but also regional. However, this inconsistency may be related to cultural, economic, religious and regional differences in the treatment of older people, the position of older people and the care of older people between different regions and nations [61].

The results also revealed that the lack of social support reduced the chance of financial maltreatment by 8%, which is not in line with the results of some studies [21, 40, 52]; however, they were consistent with the findings of a study [24]. A possible explanation for this consistency is that it investigated the relationship among social support, social network size, and financial exploitation. Moreover, higher levels of perceived social support were associated with a reduced risk of Financial Exploitation, whereas those with large non-family networks along with low perceived social support were most at risk. Therefore, the encouragement of social network expansion through “making new friends” should be de-emphasized until it is ensured that these new network members support older adults.

The lack of social support also increased the chances of psychological maltreatment by 55%, which was consistent with the results of some studies [38, 49, 60]; however, it was not in line with the findings of other studies [54]. One of the reasons for this relationship is that social support may act as a protector against stressful situations [38]. Additionally, the elderly who have been subjected to domestic violence have weak emotional relationships with their close social network (including relatives) and also suffer from a lack of social relationships [54]. It is worth noting that age, chronic diseases, social support, and depression are factors related to emotional abuse in this group of people [49]. Studies from developing countries show that living in a joint family system does not reduce loneliness and that the quality of social support networks is important in assessing the impact on elder abuse [62], also that the higher risk of elder abuse (independent type) is closely related to the emotional atmosphere among family members and the lack of intergenerational solidarity [54].

Another result revealed that the lack of social support increased the chance of physical abuse by 31%, which had the most consistency with the findings of some studies [47] and the least similarity to the results of other studies [56, 60]. Regarding physical violence, a set of predictors, including previous exposure to a traumatic event, weak social support, and limitations in doing daily life activities are of significant importance [60]. As with social support, seniors who had a positive view of community trust were less likely to experience physical or psychological abuse. Moreover, the relationship between social capital and maltreatment shows that the elderly who received a certain type of social support (instrumental support) were less exposed to physical and psychological abuse [47]. Older men are more likely to be victims of physical abuse by an intimate partner than women [56].

According to the analysis of studies [40, 41, 54, 60], the lack of social support increases the chance of neglect by 2.02 times, and they reported almost the same results. During the COVID-19 pandemic, the high prevalence of self-neglect due to the social distancing strategy and strict quarantine policies limited the access of the elderly to social support structures [41]. Additionally, elderly individuals from minority groups (racial/ethnic) were more likely to be neglected than white people [60]. Another explanation is that it is somewhat difficult to identify or even define neglect since instead of maltreatment, the offender fails to perform the appropriate action [40], and people with high emotional or social loneliness are significantly more victims of psychological violence and neglect [54].

Considering the relationship between social support and the dimension of sexual maltreatment, due to the limited number of studies, a meta-analysis was not conducted. Among the reasons for not reporting sexual abuse, it can be acknowledged that these victims may see themselves as dependent on the abuser, which makes it difficult or basically impossible for them to leave the abusive relationship [63] or because of the fear of disclosure, they do not report it [60]. Furthermore, it can also be mentioned that only previous experiences of traumatic events and low social support predicted sexual abuse [40].

One of the limitations of the study is the lack of studies using tools aimed at evaluating the aspects of social support and its relationship with the risk of elder maltreatment. Accordingly, to understand the relationship, it was attempted to assess other phenomena in other fields and evaluate the theories deeply and in more detail. In addition, although our comprehensive search strategy identified many relevant studies, most of the studies included in the meta-analysis were from high-income countries. The results may not be representative of all potential studies regarding social support and maltreatment in elderly because of the search strategy have been limited to studies published in English.

## Conclusion

To the best our knowledge, this work is the first systematic review and meta-analysis that aimed to clarify the relationship between social support and elder abuse. Overall, the important contribution of our results from previous findings showed that low levels of social support are likely to report high levels of all types of maltreatment (except for financial abuse). It seems that the evidence obtained in the studies and the definition of targeted policies are of crucial importance for decision-makers and old age. Investment in the design and implementation of social support interventions are effective components in preventing all types of elder abuse and should be considered a public health priority.

### Supplementary Information

**Additional file 1.**

## Data Availability

All data generated or analyzed during this study are included in this published article.
