# Supplementary material for: The relationship between social support and dimensions of elder maltreatment: a systematic review and Meta-analysis
Source: BMC Geriatr. 2023 Dec 18;23:869. doi: 10.1186/s12877-023-04541-6 (PMC10726566; doi:10.1186/s12877-023-04541-6)
Supplement: Supplementary file 1 — Additional file 1. [file 12877_2023_4541_MOESM1_ESM.docx]

**Appendix 1.** Search strategies used for finding methods articles. Number of retrieved articles is given in the right-hand column.

| **MEDLINE (PubMed)** |  | **684** |
| --- | --- | --- |
| **Social support**: "Social support" OR "emotional support" OR "Instrumental support" OR "information support" OR "social networking" OR "Evaluation support" AND  **Dimensions of abuse**: "neglect" OR "abuse" OR "maltreatment" OR "mistreatment" OR "violence" OR "material abuse" OR "psychological abuse" OR "sexual abuse" OR "financial abuse" OR "emotional abuse" OR "Verbal violence" OR "physical abuse" OR "mental abuse" OR "elder abuse" AND  **Elderly**: "aged" OR "aging" OR "elderly" OR "geriatric" OR "senior" OR "old age" OR "aged" OR "older" OR "older people" OR "older adults" OR "elder" OR "pensioner" OR "senile" OR "senescent" |  |  |
| **Web of Science** |  | **827** |
| #24 AND #23 AND #22  #12 OR #13 OR #14 OR #15 OR #16 OR #17 OR #18 OR #19 OR #20 OR #21  #6 OR #7 OR #8 OR #9 OR #10 OR #11  #1 OR #2 OR #3 OR #4 OR #5  TS=(neglect )  TS=("material abuse")  TS=("psychological abuse")  TS=("sexual abuse")  TS=("financial abuse")  TS=("emotional abuse")  TS=("Verbal violence")  TS=("physical abuse")  TS=("mental abuse")  TS=("elder abuse")  TS=("social networking")  TS=("social support")  TS=("emotional support")  TS=("Instrumental support")  TS=("information support")  TS=("Evaluation support")  TS=("older adults")  TS=(aged)  TS=(aging)  TS=(elderly)  TS=("older age") | 24  23  22  21  20  19  18  17  16  15  14  13  12  11  10  9  8  7  6  5  4  3  2  1 | 827  237973  123046  4115236  204528  7  1409  28458  373  3619  548  10150  70  3184  21644  91818  10064  1586  2599  229  174255  3876550  3876550  355926  52136 |
| **SCOPUS** |  | **922** |
| **Social support**: ( ( TITLE-ABS-KEY ( "Evaluation support" ) )  OR  ( TITLE-ABS-KEY ( "information support" ) )  OR  ( TITLE-ABS-KEY ( "Instrumental support" ) )  OR  ( TITLE-ABS-KEY ( "emotional support" ) )  OR  ( TITLE-ABS-KEY ( "social support" ) )  OR  ( TITLE-ABS-KEY ( "social networking" ) ) )  AND  **Dimensions of abuse:**  ( ( TITLE-ABS-KEY ( neglect ) )  OR  ( TITLE-ABS-KEY ( "elder abuse" ) )  OR  ( TITLE-ABS-KEY ( "mental abuse" ) )  OR  ( TITLE-ABS-KEY ( "physical abuse" ) )  OR  ( TITLE-ABS-KEY ( "Verbal violence" ) )  OR  ( TITLE-ABS-KEY ( "emotional abuse" ) )  OR  ( TITLE-ABS-KEY ( "financial abuse" ) )  OR  ( TITLE-ABS-KEY ( "sexual abuse" ) )  OR  ( TITLE-ABS-KEY ( "psychological abuse" ) )  OR  ( TITLE-ABS-KEY ( "material abuse" ) ) )  AND  **Elderly:** ( ( TITLE-ABS-KEY ( "older age" ) )  OR  ( TITLE-ABS-KEY ( elderly ) )  OR  ( TITLE-ABS-KEY ( aging ) )  OR  ( TITLE-ABS-KEY ( aged ) )  OR  ( TITLE-ABS-KEY ( "older adults" ) )  OR  ( TITLE-ABS-KEY ( senescent ) )  OR  ( TITLE-ABS-KEY ( senile ) )  OR  ( TITLE-ABS-KEY ( pensioner ) )  OR  ( TITLE-ABS-KEY ( elder ) )  OR  ( TITLE-ABS-KEY ( "older people" ) )  OR  ( TITLE-ABS-KEY ( geriatric ) )  OR  ( TITLE-ABS-KEY ( senior ) ) ) View less |  |  |
